# *Aeromonas veronii*-induced septic arthritis of the hip in a child with acute lymphoblastic leukemia

**DOI:** 10.1515/biol-2022-1042

**Published:** 2025-03-28

**Authors:** Yuan Tian, Xuying Yang, Ying Yang, Tianwei Lin, Guoxia Wang, Ye Zhang, Haibin Wu, Juan Wang

**Affiliations:** Department of Surgical Intensive Care Unit, Affiliated Children’s Hospital of Xi’an Jiaotong University, Xi’an, 710300, Shaanxi, China; Department of Scientific Affairs, Hugobiotech Co., Ltd, Beijing, 102600, China; Shaanxi Institute of Pediatric Diseases, Affiliated Children’s Hospital of Xi’an Jiaotong University, Xi’an, Shaanxi, China

**Keywords:** *Aeromonas veronii*, metagenomic next-generation sequencing, septic arthritis of the hip, acute lymphoblastic leukemia

## Abstract

Septic arthritis of the hip (SAH) is a prevalent form of infectious arthritis in children that can lead to serious complications if not promptly diagnosed and treated. A 6-year 4-month-old female child with a 1-year history of acute lymphoblastic leukemia chemotherapy was admitted to our hospital due to a 1-day fever. After 1 week, the child experienced right inguinal pain and exhibited severe restriction in the flexion of the right lower limb and hip. Consequently, edema was observed in the right lower extremity and foot. SAH was initially diagnosed using computed tomography and magnetic resonance imaging examinations of both hip joints. Subsequently, incision and irrigation procedure were performed on the hip joint. Following the surgery, pus metagenomic next-generation sequencing (mNGS) were conducted promptly, and the mNGS analysis indicated an *Aeromonas veronii* infection. The diagnosis of *A. veronii* SAH was subsequently confirmed through polymerase chain reaction. The child’s condition was successfully treated with a combination of amikacin and imipenem–cilastatin, leading to improvement and subsequent discharge in a satisfactory state. SAH caused by *A. veronii* is a rare occurrence, and the utilization of mNGS holds significant potential for the early detection of uncommon infections in immunosuppressed children.

## 1 Background

*Aeromonas veronii*, a Gram-negative, rod-shaped, and facultative anaerobic bacterium, is widely prevalent in aquatic environments [1]. As a noteworthy emerging zoonotic pathogen originating from aquatic sources, it can induce various ailments in immunosuppressed individuals, including gastroenteritis, skin and soft tissue infections, bacteremia, and even acute septic arthritis [2–4]. Between 32 and 40% of septic arthritis cases in children pertain to the hip joint, a prevalent and potentially severe condition primarily caused by bacterial agents [5]. While *Staphylococcus aureus* is the primary pathogen, other microorganisms with pathogenic potential, such as *Escherichia coli*, *Salmonella enterica serovar Enteritidis*, *Group B Streptococcus* are also implicated [6]. However, there is a lack of documented cases of septic arthritis of the hip (SAH) caused by *A. veronii*, which still has limited knowledge regarding the clinical presentation and treatment. Only three instances of arthritis caused by *A. veronii* in adults have been documented to date [2,7,8]. There have been no reported cases of SAH caused by *A. veronii* in children. Here, we report the first case of SAH due to *A. veronii* infection in a female child with acute lymphoblastic leukemia (ALL) that was confirmed by metagenomic next-generation sequencing (mNGS).

## 2 Case presentation

A 6-year 4-month-old female child visited to our hospital with a 1-day history of fever on 28 September 2023. One day before presentation, the child had fever and chills with no obvious inducement and the highest body temperature was 39.7°C. Despite receiving oral ibuprofen at home, the child continued to have recurrent fever. Upon evaluation in the emergency department, infection-related markers indicated a hypersensitive C-reactive protein (hs-CRP) level of 9.85 mg/L (normal range: 0–3 mg/L). Consequently, the child received empiric treatment consisting of a single dose (1.5 g) of cefoperazone and sulbactam sodium injection and was subsequently admitted to the hospital (on 29 September 2023). The child was diagnosed with ALL in December 2022 at our hospital, and has been undergoing chemotherapy treatment following the vincristine, daunorubicin, l-asparagine, and prednisone regimen up until the present time.

Upon admission, the physical examination revealed that the child was conscious but exhibited a diminished mood and pharyngeal congestion, while the examinations of the nervous system, cardiopulmonary system, and abdominal region showed no notable abnormalities. The routine blood test conducted upon admission on 29 September indicated a white blood cell count of 0.07 × 10^9^/L (normal range: 4.3–11.3 × 10^9^/L), a hemoglobin level of 96 g/L (normal range: 118–156 g/L), a platelet count of 66 × 10^9^/L (normal range: 167–453 × 10^9^/L), the lymphocyte and neutrophil count were not detected. Hs-CRP and procalcitonin levels were measured at 11.17 mg/L and 20.96 ng/mL (normal range: 0.00–0.05 ng/mL), respectively. The G test, galactomannan test, blood culture, and respiratory viral polymerase chain reaction (PCR) results all yielded negative findings. Chest computed tomography (CT) showed no obvious abnormalities. Empirical anti-infective treatment was initiated with intravenous imipenem–cilastatin sodium (0.6 g, q 6 h), platelets were replenished through blood transfusion, and subcutaneous granulocyte colony-stimulating factor was administered to stimulate granulocyte production upon admission. After 3 days of hospitalization, the child continued to experience fever with a maximum body temperature of 40.0°C. In order to address the infection, the administration of vancomycin hydrochloride (480 mg, q12h) was initiated; however, the therapeutic response remained unsatisfactory.

**Figure 1:**
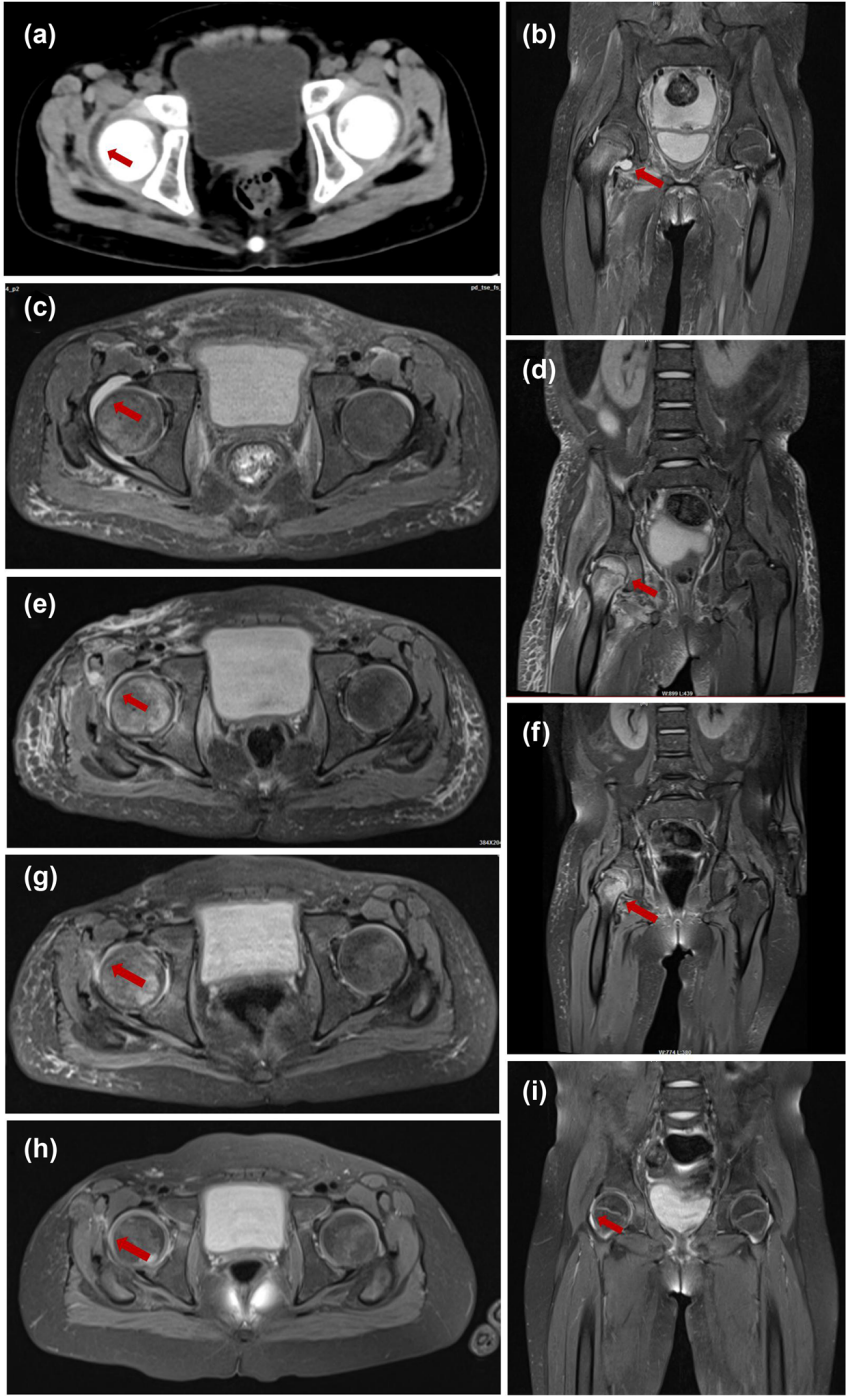
Radiograph check-up examinations. (a) Initial CT of bilateral hip joints showing aspiration of right hip joint effusion. (b) and (c) Initial MRI of bilateral hip joints showing marrow edema of right proximal femur and aspiration of right hip joint effusion. (d) and (e) Repeat MRI of bilateral hip joints on the 15th day after surgery showing a decrease in right hip capsule effusion compared to previous scans. (f) and (g) Repeat MRI of bilateral hip joints on the 26th day after surgery showing a further reduction in right hip capsule effusion and a significant reduction of soft tissue swelling. (h) and (i) Hip MRI at 1 month after discharge revealed a significant reduction in right hip capsule effusion and a marked decrease in the size of soft tissue edema. Red arrows indicate the target lesion.

**Figure 2:**
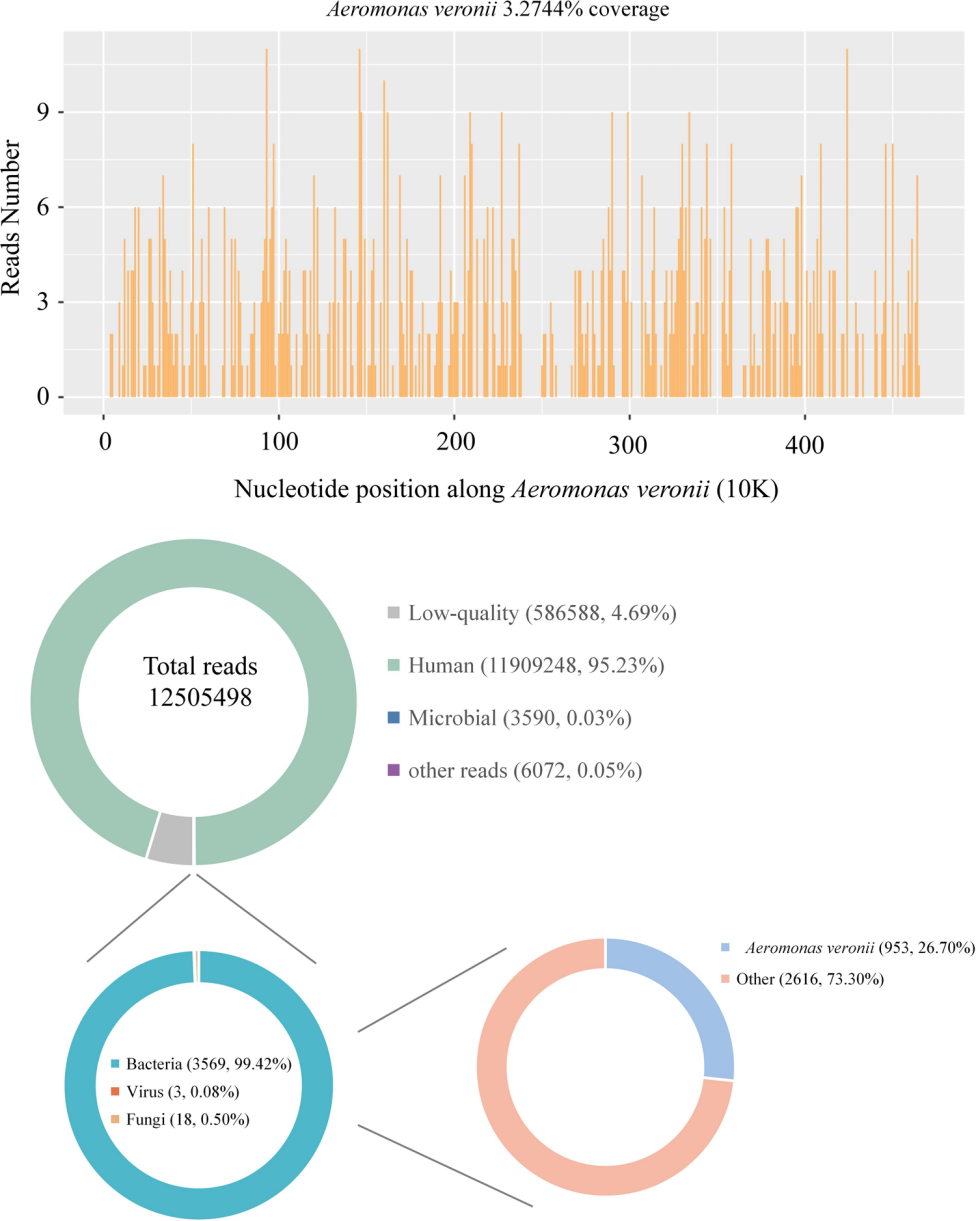
*A. veronii* was detected by mNGS.

On the sixth day of admission, the child presented with right inguinal pain and the presence of multiple lymph nodes, approximately the size of mung beans, were palpable in the right inguinal area. Based on the results of a superficial lymphadenitis assessment using right inguinal color ultrasound, the child’s treatment regimen was adjusted to include a combination of vancomycin hydrochloride (480 mg, q12h) and imipenem–cilastatin sodium (0.6 g, q6h) for anti-infections. One week post-admission, the child continued to experience recurrent fever. Routine blood testing indicated myelosuppression following chemotherapy, prompting the addition of voriconazole (96 mg, q12h) for anti-infective treatment. Despite these interventions, the fever persisted and the child developed worsening right inguinal pain, accompanied by severe limitations in right lower limb flexion and right hip motion. Additionally, edema was observed in the right lower extremity and foot. CT and magnetic resonance imaging (MRI) examinations of bilateral hip joints revealed SAH in the right region (Figure 1a–c). Based on the findings of a bilateral inguinal color ultrasound, it was determined that the child was not a suitable candidate for hip aspirations. Following a multidisciplinary consultation, it was ultimately advised that she undergo an incision, irrigation, and drainage procedure on the right hip, under general anesthesia, scheduled for 11 October 2023. The examination of the joint cavity revealed a substantial amount of necrotic tissue, and a drainage of 1 mL of yellowish pus was performed from the right hip. The collected joint pus was subsequently submitted for PACEseq mNGS analysis. Detailed method of mNGS is described in the supplementary methods. The results of mNGS analysis conducted after 24 h revealed the presence of *A. veronii*, with a total of 953 unique reads (Figure 2). Further confirmation of *A. veronii* was obtained through PCR testing of the pus (Figures S1 and S2a and b), with additional details provided in the supplementary methods. Based on these findings, the child was diagnosed with SAH caused by *A. veronii*. Consequently, the administration of empiric vancomycin hydrochloride was discontinued, and a combination of amikacin (0.15 g, q12h) and imipenem/cilastatin was introduced for anti-infective therapy.

Following the aforementioned treatment, the child’s body temperature returned to normal on the sixth day after the surgical procedure. She did not report any complaints of right inguinal pain while under bed rest. Additionally, the edema in the right lower extremity and foot showed signs of improvement. A repeat bilateral hip MRI on the 15th day after surgery indicated a further decrease in right hip capsule effusion compared to previous scans (Figure 1d and e). The child’s body temperature remained stable, leading to the discontinuation of amikacin and its replacement with ceftazidime for anti-infection treatment. On the 26th postoperative day, she could walk independently, however, with a limp in the right lower limb. Subsequent bilateral hip MRI revealed a further reduction in right hip capsule effusion (Figure 1f and g), prompting the continuation of chemotherapy with the previously prescribed regimen. On the 33rd postoperative day, blood routine and infection indexes were all normal, and she was discharged on the postoperative Day 34. After she was discharged oral cefdinir dispersible tablets administration was continued (75 mg, tid 4 weeks).

**Figure 3:**
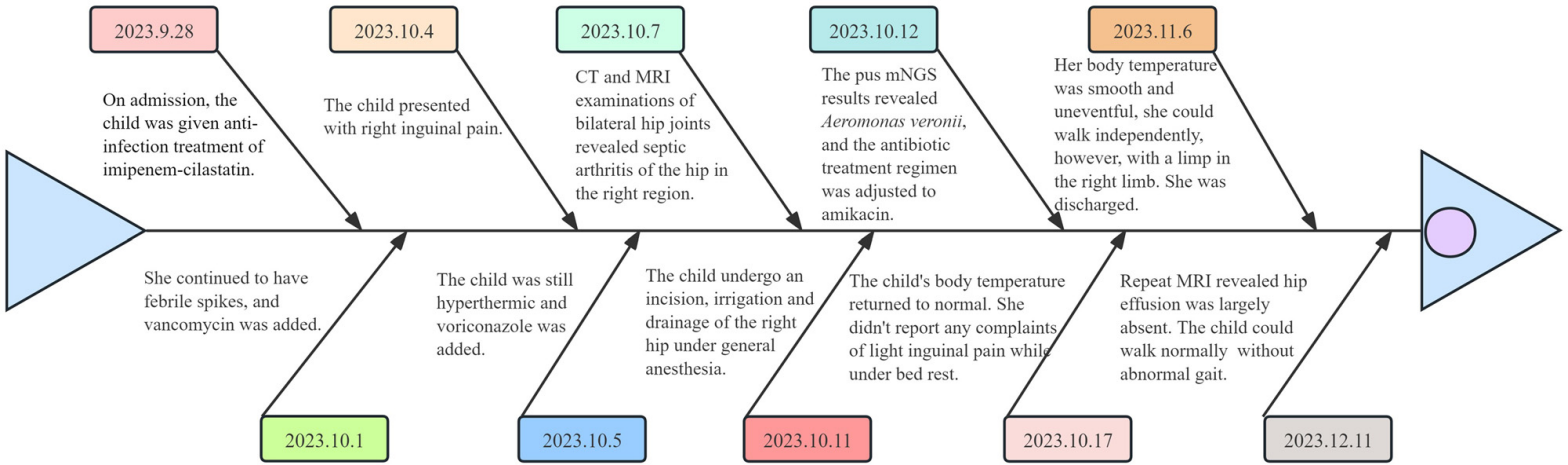
Timeline of the condition diagnosis.


One month after discharge, the child could walk and squat normally without abnormal gait. Hip MRI revealed a significant reduction in right hip capsule effusion and a marked decrease in the size of soft tissue edema (Figure 1h and i). The clinical progression is depicted in Figure 3.

**Informed consent:** Informed consent has been obtained from all individuals included in this study.
**Ethical approval:** The research related to human use has been complied with all the relevant national regulations, institutional policies and in accordance with the tenets of the Helsinki Declaration, and has been approved by the Ethical Review Committee of Affiliated Children’s Hospital of Xi’an Jiaotong University (2023-002-02).

## 3 Discussion and conclusion

To the best of our knowledge, the occurrence of SAH caused by *A. veronii* is exceedingly rare, with minimal prior studies documenting such cases. In this study, we present the first document case of *A. veronii* identified as the causative pathogen in joint pus in a female child with ALL, detected by mNGS and confirmed by PCR.

SAH is a common septic joint condition in children and, if untreated, can lead to severe complications [5,9]. Clinical signs of SAH may include fever, hip pain, and restricted range of motion [10,11], though early diagnosis can be challenging due to the lack of typical symptoms. In this case, the child initially presented with fever, followed by right inguinal pain on Day 6, and typical SAH symptoms only appeared a week after admission. Consequently, SAH was not initially suspected. The primary cause of septic arthritis in children is typically hematogenous spread, with the hip joint being particularly susceptible [11]. In this case, the child had no signs of bloodstream infection, and blood cultures were negative. Additionally, there was no recent trauma, skin ulceration, or seafood consumption, leaving the infection source unclear.

*S. aureus* is the predominant organism commonly associated with SAH, and there has been a notable rise in the prevalence of methicillin-resistant *S. aureus* in recent times [12]. *Kingella kingae* and *Streptocococcus* species constitute the majority of the remaining cultured organisms [13]. Over the past few years, an increasing number of untypical pathogens have been identified in SAH. For instance, a case study involving a young boy diagnosed with hemophagocytic lymphohistiocytosis revealed fungal hip arthritis, indicating the need to consider underlying immunologic deficiencies and atypical infectious causes of septic arthritis [14]. However, according to existing literature, blood and joint fluid cultures frequently yield negative results, with a reported false-negative rate of 78% for Gram-stain microscopy in cases of septic arthritis [15]. Specifically, the Gram stain revealed the presence of bacteria in only 4% of cases, while the fluid culture yielded positive results in 17% of cases, thereby complicating the diagnosis and treatment process [16].

mNGS enables comprehensive and highly sensitive identification of pathogens, independent of traditional culture methods. Previous studies have demonstrated that the positive rate of mNGS (69.1%) is higher than culture (45.2%) and usual care (culture + PCR) (57.1%) in pediatric osteoarticular infections [17], and the accuracy of mNGS was significantly higher at 71.2% compared to the culture at 39.0% [18]. In our clinical setting, mNGS has been routinely employed for pathogen detection in critically ill patients, particularly among immunosuppressed pediatric individuals. In this particular instance, the child exhibited a 1-year duration of ALL and had received chemotherapy treatments, rendering them susceptible to various opportunistic infections. The identification of *A. veronii* through pus mNGS in the child was not surprising. Nevertheless, mNGS also has limitations, interpreting mNGS results is challenging and often requires assistance from mNGS laboratory experts, and reported results may not reflect the patient’s true infectious status. In addition to interpretation challenges, the expense and turnaround time of commercial mNGS must be considered. Therefore, we used the PCR method to verify the mNGS results in this case. Unfortunately, pus culture was not performed initially due to an insufficient sample size. During surgery, a diagnostic puncture of the right hip joint yielded 1 mL of yellow purulent fluid. Following this, an incision, irrigation, and drainage with tube placement were performed. Upon opening the joint capsule, significant necrotic tissue and yellowish pus were observed, and extensive irrigation with saline and iodine solution was carried out. However, the pus was contaminated with iodine, rendering it unsuitable for culture. Given the limited volume of viable sample and the high sensitivity of mNGS, the purulent fluid from the initial diagnostic puncture was sent for mNGS testing after family consent. As a result, microbiological culture and antibiotic susceptibility testing were not performed. In clinical practice, when sample collection is relatively easy and the sample size is sufficient, traditional and cost-effective diagnostic methods, such as culturing, are still preferred for pathogen identification.

*A. veronii*, classified under Proteobacteria, is recognized as a potential pathogen in aquaculture [1,19]. It is also an emerging human enteric pathogen, causing mainly gastroenteritis with various severities and also often being detected in patients with inflammatory bowel disease [20,21]. *Aeromonas* spp. infections particularly extra-intestinal infections have increased in frequency recently. Previous study suggested that *Aeromonas* spp. has an important role in pediatric practice and requires appropriate attention and monitoring [21]. To date, there have been no reported cases of SAH caused by *A. veronii* in children. Our case study suggests that *A. veronii* may serve as a potential causative agent in immunosuppressed children.

Potential sequelae of SAH encompass chondrolysis, avascular necrosis of the femoral head, and growth disturbance of the proximal femur. These complications have the potential to lead to profound disability, thereby potentially requiring reconstructive intervention in due course [22,23]. Timely identification and treatment can effectively prevent significant complications. The therapeutic approaches for SAH include surgical intervention and antibiotic therapy. Drainage of SAH can be achieved through arthrocentesis, arthroscopy, or arthrotomy. Arthrocentesis and arthroscopy are effective choices for the treatment of SAH, arthrotomy might be associated with inferior clinical outcomes and more radiological sequelae [24,25]. However, in this particular case, arthrocentesis was not a viable option due to the significant thickening of the hip synovium. Fortunately, the child did not experience any sequelae or complications following surgery. Retrospective study demonstrated all Aeromonas species isolates were uniformly susceptible to cefepime, amikacin, azithromycin, and meropenem [26], and the amikacin along with imipenem showed good penetration into bone and joint tissues reaching concentrations exceeding the MIC90 and/or MIC breakpoints of common bone and joint infections pathogens [27]. Therefore, amikacin combined with imipenem/cilastatin were selected to as anti-infective agents in our case. Subsequently, the patient experienced a favorable outcome and remained asymptomatic during the follow-up period. The diagnosis and treatment of this case can serve as a reference for similar cases in the future.

Our case report serves to emphasize the occurrence of a rare case of SAH in an immunosuppressed child caused by *A. veronii*, thereby highlighting the importance of pediatricians’ awareness of such cases. Furthermore, mNGS emerges as a potent diagnostic tool for immunosuppressed children with uncommon infections, effectively preventing missed or incorrect diagnoses.

## Supplementary Material

Supplementary material
