# Supplementary material for: Aeromonas veronii-induced septic arthritis of the hip in a child with acute lymphoblastic leukemia
Source: Open Life Sci. 2025 Mar 28;20(1):20221042. doi: 10.1515/biol-2022-1042 (PMC11964183; doi:10.1515/biol-2022-1042)
Supplement: Supplementary material [file biol-2022-1042-sm.pdf]

Supplementary material

S1 Methods

S1.1 mNGS method

The joint pus was sealed aseptically on dry ice to Hugobiotech Co., Ltd., (Beijing, China) to perform mNGS detection immediately. The DNA of the samples were extracted using the PACEseq DNA Extraction Reagent Kit (YGZZ014; HugoBiotech, Beijing, China). DNA concentration and quality were checked through Qubit 3.0 Fluoremeter (Invitrogen, Q33216) and agarose gel electrophoresis (Major Science, UVC1-1100). Subsequently, the DNA sequencing libraries were constructed using the PACEseq Universal DNA Sequencing Library Prep Kit (YGZZ005; HugoBiotech, Beijing, China). Library quality control was performed by Qubit 3.0 Fluoremeter (Invitrogen, Q33216) and Agilent 2100 Bioanalyzer (Agilent Technologies, Palo Alto, USA). Qualified DNA libraries with different barcode tags were pooled and then sequenced using the Illumina Nextseq 550 sequencing platform (Illumina, San Diego, USA) and a SE75bp sequencing strategy. After obtaining the sequencing data, high quality data was generated by filtering out connectors, low quality, low complexity and shorter sequences. Next human-derived sequences matching to the human reference database (hg38) were removed by using SNAP software. The remaining data were then aligned to the microbial genome database using Burrow-Wheeler Alignment. This database contains a large collection of microbial genomes from NCBI containing more than 30,000 microorganisms, including 17,748 species of bacteria, 11,058 species of viruses, 1,134 species of fungi, and 308 species of parasites. The microbial composition of the samples was finally determined. The positive criteria for the mNGS result were set as follows: For bacteria other than TB, fungi other than Cryptococcus and parasites: sequencing coverage in the top 10 of all pathogens detected and not detected in the negative control (NTC); or sample/NTC with an RPM (reads per million mapped reads) ratio greater than 10.

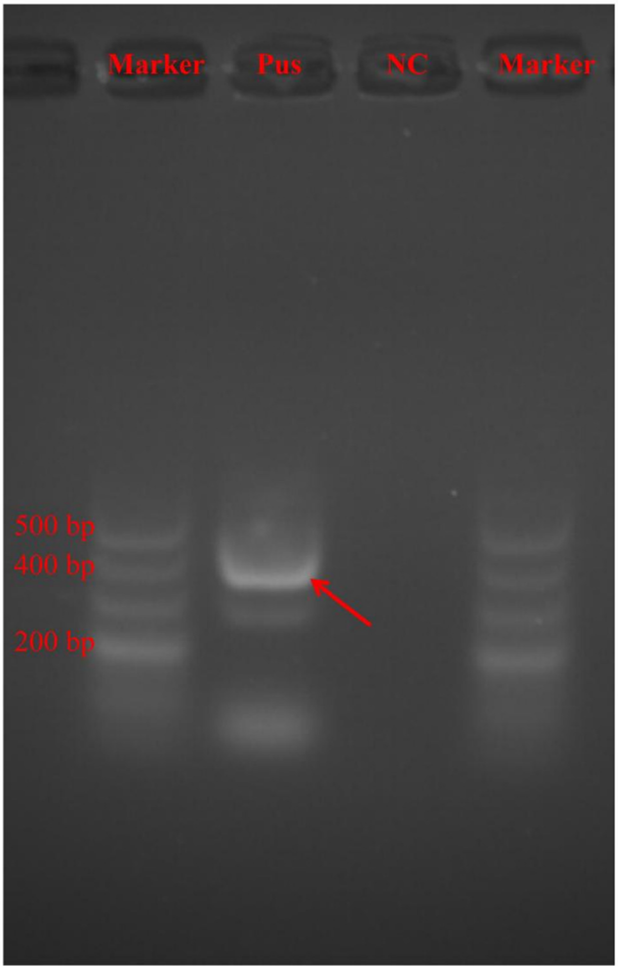

Figure S1: PCR products were separated by gel electrophoresis on a 1.5% agarose gel. The red arrow indicates the target band of PCR amplification.

S1.2 PCR method

PCR was utilized for *Aeromonas veronii* nucleic acid detection. DNA was extracted from joint pus using a DNA extraction Mini Kit (56304; Kaijie). All amplifications were performed using SU PCR Mix (NH9329; nuhighbio.com) and in an Eppendorf Mastercycler gradient PCR thermal cycler (Brinkmann Instruments). In the PCR reaction, the target gene fragment was amplified with 5-*CCTATGGCCTGAGCG AGAAG*-3 and 5-*CCAGTTCCAGTCCCACCACT*-3 as the upstream and downstream primers, respectively, to give a 424-bp fragment. The product of PCR was confirmed by Sanger sequencing.

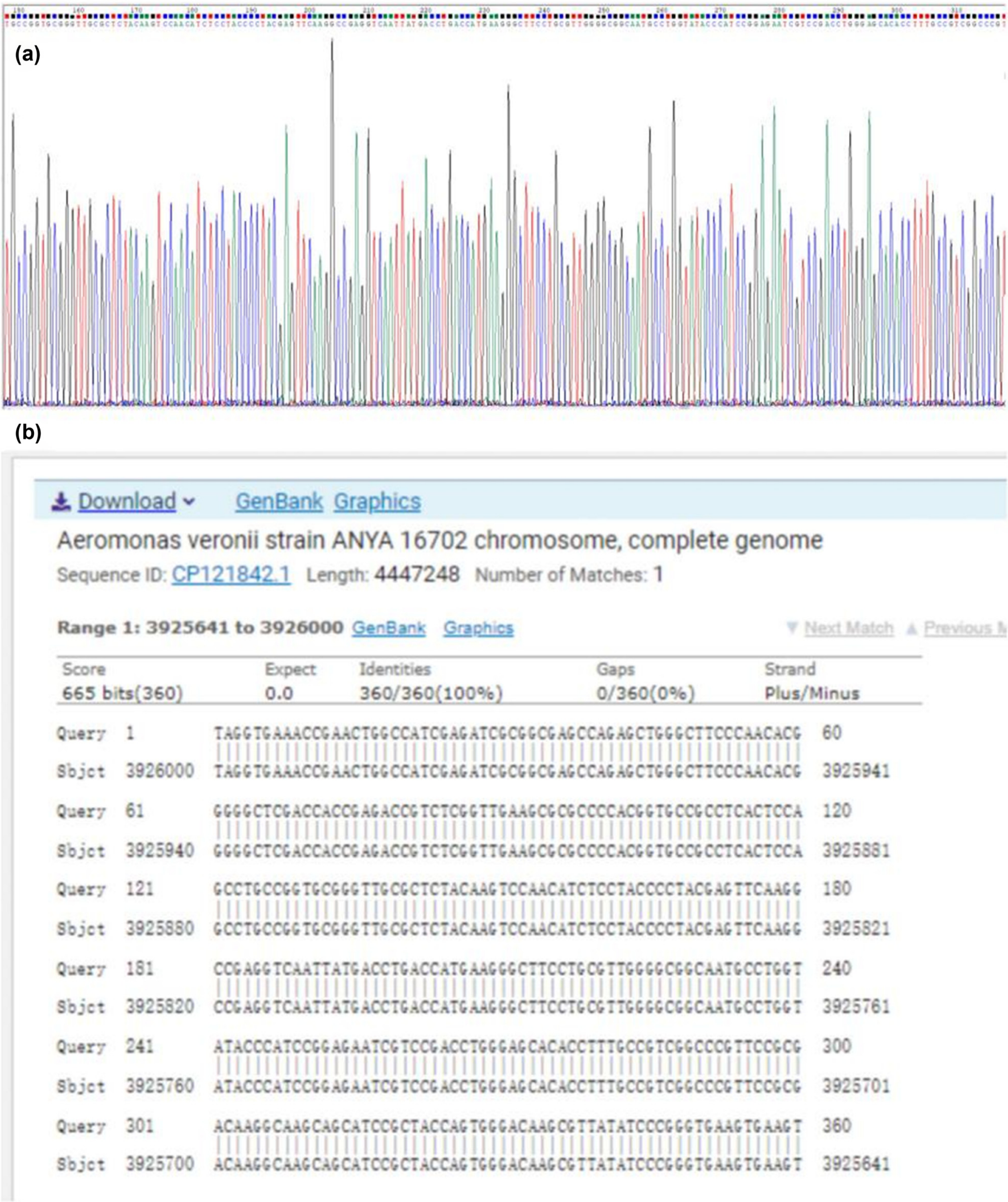

Figure S2: Results of the Sanger sequencing. (a) Sanger sequencing peak map. (b) The benchling alignment of the Sanger sequencing result to the reference sequence of *Aeromonas veronii*.
